# Knowledge of nursing professionals about the antiseptics used in patient care with bladder catheters

**DOI:** 10.1186/2197-425X-3-S1-A930

**Published:** 2015-10-01

**Authors:** MR Silva, MDCF Araujo, GC Santos, EC Santos, T Figueiredo, DA Barbosa

**Affiliations:** Universidade Estadual de Santa Cruz, Health Sciences, Ilhéus, Brazil; Universidade Federal de São Paulo, Health Sciences, São Paulo, Brazil; Base Hospital Luis Eduardo Magalhães, Nursing, Itabuna, Brazil

## Introduction

Antisepsis is of utmost importance for the prevention and control of nosocomial infections, given that they have the purpose of reducing or inhibiting the growth of microorganisms on the skin and mucous.

## Objectives

o identify the knowledge of nursing professionals involved in patient care carrier bladder catheter.

## Methods

A descriptive, exploratory study, quantitative performed in a hospital in the southern region of Bahia-Brazil. Data collection between January and March 2014. Study population constituted by nursing professionals working directly with care in the intensive care unit. Ethics committee protocol and the Federal University of São Paulo: 319 255/2013.

## Results

The questionnaire was answered 32 professionals. Participants: 28 women (87.5%). Predominant age group: 30 to 39 years 26 (81.25%). Experience of time in intensive care: 1 to 5 years: 21 (65.6%). Catheters average of 5 per week. Knowledge of the protocols on indwelling catheters: 27 (84.4%). Indications for catheterization and choosing the appropriate antiseptic for catheter insertion: 28 (87.5%). Daily care of the urinary meatus and perineal region: 29 (90.6%). Using the ideal antiseptic for collecting urine tests: 30 (93.75).

## Conclusions

The professionals have knowledge about the correct use of antiseptics in the prevention and surveillance of urinary tract infections associated with indwelling catheters. Are routinely made permanent education activities, supervision and update the rules and routines related to monitoring and control measures.

## Grant Acknowledgment

The knowledge about the correct use of antiseptics is a major action against acquiring infections in the hospital environment, showing the intensive care unit, where many invasive procedures are performed. Adopt basic preventive measures such as education and encouraging health professionals about the importance of this act is essential for the occurrence of these infections decrease.
